# Institution Publication Feature Analysis Based on Time-Series Clustering

**DOI:** 10.3390/e24070950

**Published:** 2022-07-07

**Authors:** Weibin Lin, Mengwen Jin, Feng Ou, Zhengwei Wang, Xiaoji Wan, Hailin Li

**Affiliations:** 1 College of Business Administration, Huaqiao University, Quanzhou 362021, China; lwb@qztc.edu.cn (W.L.); 202022011064@mail.bnu.edu.cn (M.J.); oufeng@stu.hqu.edu.cn (F.O.); 1916411037@stu.hqu.edu.cn (Z.W.); wanxiaoji@hqu.edu.cn (X.W.); 2 TSL Business School, Quanzhou Normal University, Quanzhou 362021, China; 3 Research Center of Applied Statistics and Big Data, Huaqiao University, Xiamen 361021, China

**Keywords:** institution publication, feature analysis, time series clustering, numerical values, trend analysis, associated network

## Abstract

Based on the time series of articles obtained from the literature, we propose three analysis methods to deeply examine the characteristics of these articles. This method can be used to analyze the construction and development of various disciplines in institutions, and to explore the features of the publications in important periodicals in the disciplines. By defining the concepts and methods relevant to research and discipline innovation, we propose three methods for analyzing the characteristics of agency publications: numerical distribution, trend, and correlation network analyses. The time series of the issuance of articles in 30 important journals in the field of management sciences were taken, and the new analysis methods were used to discover some valuable results. The results showed that by using the proposed methods to analyze the characteristics of institution publications, not only did we find similar levels of discipline development or similar trends in institutions, achieving a more reasonable division of the academic levels, but we also determined the preferences of the journals selected by the institutions, which provides a reference for subject construction and development.

## 1. Introduction

Time series are groups of sequence data arranged in time order, which are composed of historical values and are important data sources. Through time series analysis, the future development trend of related problems can be predicted; this method is widely used in the field of economy, finance, environment, climate, production, and life [1,2]. Clustering is a data-mining algorithm that divides data objects with similar characteristics into clusters based on a measurement of similarity, and divides dissimilar objects into different clusters. Time-series clustering is a common method used for studying time-series characteristics. According to different objects, time-series clustering can be divided into full-sequence, subsequence, and time-point clustering. Common clustering algorithms include partition-, hierarchical-, density-, grid-, and model-based clustering algorithms [3,4]. Time-series clustering can be applied for the analysis of multiple problems. For example, Pierpaolo [5] clustered a time series and applied the clustering results to monitor air pollution. The data obtained from the number of documents issued by institutions for several years constitute time-series data, that is, the time series data of institutions’ output.

Under the background of China’s double-first-class construction [6], scientific research papers and achievements have become criteria for evaluating first-class universities and disciplines, so universities are paying more attention to the quality and quantity of scientific research across various disciplines. Double first-class refers to “first-class universities and disciplines in the world”, which is a major strategic decision made by China to improve China’s education level and international competitiveness. Universities encourage scholars to publish articles in high-quality journals. Therefore, the scientific research level of institutions is closely related to the number of papers published by scholars in high-quality journals [7]. To measure the development level of universities in a more scientific manner, the publishing situation of universities in relevant academic journals should be considered from the perspective of scientific research achievements.

At present, the main directions of the institutions that are publishing papers include summarizing the characteristics of the relevant institutions’ publications, discussing the scientific research level or the subject evaluation of institutions based on bibliometric methods, and analyzing the characteristics of institutional cooperation networks. Some scholars have evaluated the scientific research performance of universities according to the publication citations, and have analyzed the academic impact of universities [8,9]. Muhuri [10] analyzed the citation structure of specific journals, built an author co-citation network, and studied the relationship between publications and the nationality of authors. In addition, a few scholars have discussed such matters from the perspective of the relationship between institutions, colleges, and journals. National policies also impact the scientific research level of universities. Under the influence of national policies, the scientific research level of universities can be calculated from the bibliometric analysis of publications [11]. The journals founded by institutions scientifically contributed to these institutions, and such papers have multiple features, which can be indirectly used to measure the scientific research ability of the institutions [12]. Through social network analysis and text mining, Zhiya Zuo [13] studied the relationship between university researchers and publications, and found that multidisciplinary cooperation might not achieve the desired results. Jiang Xia [14] analyzed the characteristics o the articles and authors of college journals from the perspective of the number of published papers, downloads, and citations, and put forward suggestions for the development of college journals.

Most of the existing studies have used bibliometric analysis methods to explore the characteristics and disciplinary development of institutional publications based on a variety of types of publication data, but there is a lack of research on the methods of analyzing publications from the perspective of time series. Therefore, from the perspective of time-series clustering, we designed an method suitable for anazlying publication document data, which can be used to mine the characteristics of publications, and will help with understanding the development of university disciplines and exploring the relationship between universities and journals.

## 2. Theoretical Basis

In this section, similarity measurement and affinity propagation clustering methods are introduced to facilitate the following discussion.

### 2.1. Similarity Measurement

In data mining, it is usually necessary to calculate the distance or similarity between data objects to quantitatively reflect the relationship between them. According to the measurement results, data mining tasks, such as clustering, classification, and abnormality analysis, can be performed. In time-series data mining, similarity measurements are also commonly used to describe the similarity between two time series. Similarity measurement is one of the main tasks of time-series data mining, and the quality of the results directly affects the quality of the mining [15]. There are many similarity measurement methods, which are usually selected according to the research data. In time-series data mining, the common measurement methods include Euclidean distance and dynamic time warping (DTW) [16,17], shape-based distance (SBD) [18,19], and matrix profile distance (Mpdist) [20,21].

Among many similarity measurements, Euclidean distance is one of the most commonly used. The Euclidean distance can be used to measure the distance between data objects in multidimensional space and has a wide range of application scenarios. Suppose there are two objects, $X=\{x_{1},x_{2},\cdots ,x_{n}\}$ and $Y=\{y_{1},y_{2},\cdots ,y_{n}\}$, where dist(X,Y) denotes the Euclidean distance between them; the corresponding formula is shown in Equation (1).

(1) $$dist\left(X,Y\right)=\sqrt{\sum_{i=1}^{n}{\left(x_{i}-y_{i}\right)}^{2}}$$

The Euclidean distance is simple to operate and has low time complexity, thus is often used to measure the similarity between data objects of equal length. However, because the Euclidean distance is more sensitive to time-series outliers or data mutations, data preprocessing, such as standardization, is generally first required.

### 2.2. Affinity Propagation Algorithm

Affinity propagation (AP) is an algorithm developed by Fery et al. [22]. This semisupervised clustering algorithm based on affinity propagation was proposed in 2007. Compared with general clustering algorithms, the main advantages of the AP algorithm are that it does not need to determine the number of clusters in advance, and the cluster center is a data point in the dataset to be clustered. The given similarity matrix can be symmetric or asymmetric. The main idea of the AP algorithm is that all data objects are initially taken as potential clustering centers, a given similarity matrix is combined with a specific method for iterative calculation, and the best clustering results of the original dataset are finally obtained [23,24].

The core of the AP clustering algorithm as shown in Algorithm 1 involves updating two metrics for data point *i*: “responsibility” and “availability”. Responsibility, r(i,k), is used to describe the degree to which data point *k* is suitable as a cluster center of data point *i*, and the direction of information transmission is from *i* to *k*. Availability, a(i,k), is used to describe the degree of suitability of the data point *i* to select the data point *k* as the cluster center, and the direction of information transmission is from *k* to *i*. In addition, the AP clustering algorithm needs to input the similarity matrix *S*; when i≠k, s(i,k) can be obtained by Formula (2); when i=k, s(k,k) means self-bias (preference), which needs to be uniformly set. The larger the value, the more likely the data point will be selected as the cluster center, and the greater the final number of clusters; on the contrary, the overall number of clusters will be less. Typically, the bias is set to the median of *S*.
**Algorithm 1** Affinity propagation clustering.**Input:** similarity matrix *S*, where s(i,i) is the median value**Output:** set of clustering results1:Initialize the responsibility *R* and availability *A* to zero matrices; let the number of iterations t=12:Update the responsibility matrix *R* according to Formula (2)3:Update the availability matrix *A* according to Formula (3)4:Substitute λ into Formula (4) to update the responsibility *R* and availability *A* matrices5:Obtain the cluster center set $c=\{c_{1},c_{2},\cdots ,c_{m}\},0<m\leq n$ according to r(i,i)+a(i,i)>06:Iteratively perform Ateps (2), (3), (4), and (5) and the number of iterations at the same time t= t +1; stop the iteration if the cluster center set *c* does not change, or t≥max_Num7:According to the $\max\{a\left(i,c_{k}\right)+r\left(i,c_{k}\right)\}$ rules, the data points are assigned to the corresponding clusters, and the clustering results are combined with *C*

The first step in implementing Algorithm 1 is initializing the responsibility *R* and availability *A* to zero matrices, followed by the iterative operation. For each data point, the calculation formulas of attraction and attribution are substituted, and *R* and *A* are continuously updated according to Formulas (2) and (3). The cluster center is obtained according to the judgment formula r(i,i)+a(i,i)>0. The algorithm stops when the cluster centers converge or when the maximum number of iterations is reached.

(2) $$\begin{array}{c} r\left(i,k\right)=s\left(i,k\right)-\underset{k^{{}^{\prime }}\neq k}{max}\left\{a\left(i,k^{{}^{\prime }}\right)+s\left(i,k^{{}^{\prime }}\right)\right\};\;\;i\neq k\\ r\left(k,k\right)=s\left(k,k\right)-\underset{k^{{}^{\prime }}\neq k}{max}\left\{a\left(k,k^{{}^{\prime }}\right)+s\left(k,k^{{}^{\prime }}\right)\right\};\;i=k \end{array}$$

(3) $$\begin{array}{c} a\left(i,k\right)=\min\{0,r\left(k,k\right)\left.+\sum_{i^{{}^{\prime }}\notin \left\{i,k\right\}}\max\{0,r\left(i^{{}^{\prime }},k\right)\}\right\};\;i\neq k\\ a\left(k,k\right)=\sum_{i^{{}^{\prime }}\neq k}\max\{0,r\left(i^{{}^{\prime }},k\right)\};\;\;\;\;\;\;\;\;i=k \end{array}$$

For some special data points, the AP clustering algorithm may fail to produce reliable clustering results after multiple iterations, which is called “data shocks”. To reduce the occurrence of this phenomenon, the AP algorithm introduces a damping factor (λ) to update the weighted values of attraction belonging at each iteration. The value range of the damping coefficient is 0∼1, and the default value is 0.5. The formula of the update is shown as follows: if λ is larger, the change in the current period $R^{\left(n\right)}$ and $A^{\left(n\right)}$ relative to the previous period $R^{\left(n-1\right)}$ and $A^{\left(n-1\right)}$ is smaller, and the change in the number of clusters is also smaller.

(4) $$\begin{array}{c} R^{\left(n\right)}=\left(1-\lambda \right)*R^{\left(n\right)}+\lambda *R^{\left(n-1\right)}\\ A^{\left(n\right)}=\left(1-\lambda \right)*A^{\left(n\right)}+\lambda *A^{\left(n-1\right)} \end{array}$$

## 3. Analysis of Institutions Output Characteristics

In this section, the research framework is described, two novel indexes are defined, and three analysis methods are proposed. This section focuses on the research ideas and the process of quantifying data.

### 3.1. Research Motivation

Time-series data published by universities and colleges in important disciplinary journals often have recessive features. Mining these recessive characteristics is helpful for understanding the disciplined development of universities. The cooperative relationship between university institutions and important journals was explored, the results of which provide a reference for the discipline construction and development of university institutions. Therefore, to discover the hidden characteristics and rules of publications, a scientific and effective method was designed for the analysis of the characteristics of publications according to the time-series data of institutional publications.

**Figure 1:**
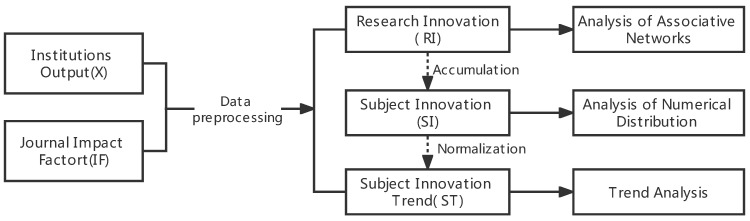
Research framework.

Based on the research on the time series of annual publications of representative academic journals by universities, we considered three aspects of publication feature analysis. The overall research framework is shown in Figure 1. First, data on the volume of papers published by the original institutions and the impact factor of the journal were collected, and data preprocessing was performed. The volume of papers published by the institutions refers to the number of papers published by a research institution in a representative journal of a certain discipline in a certain year. Second, the concepts of “research Innovation” and “subject Innovation” were proposed, and the original data were transformed into time series of the degree of research innovation and the degree of discipline innovation. Finally, from the perspective of university institutions publishing papers in important journals, three kinds of characteristic analysis, including numerical distribution, trend, and associative network analyses, were used to explore the current situation and development of university disciplines, as well as the relationship between universities and journals.

### 3.2. Related Definitions

Before data processing, two indicators were defined to measure the scientific research capability of institutions, which reflect the influence of institutions on specific subjects and on different journals.

#### 3.2.1. Research Innovation

Research innovation (RI) is the result of processing a number of published papers after considering the journal’s influence. Compared with the unconverted paper volume, RI not only reflects the influence of universities, but also enables the performance of universities in different journals to be comparable and additive. It is also convenient for subsequent research in the field of discipline development. RI conversion is achieved as follows: First, the number of papers published by institutions in specific journals is normalized. Second, combined with the compounding factor of the journal, the research innovation score of the journal can be obtained by weighted processing.

To eliminate the difference in the total number of papers published in different journals, it is necessary to normalize the number of published papers. Assuming that *m* university institutions and *n* representative journals are studied, *X* is the data matrix of the published paper quantity of university institutions in journals, where $x_{p,q}$ is the value of the publication quantity of *p* university institutions in the representative journal *q*. Normalization processing transforms matrix *X* in columns, so that the values are mapped to the interval [0, 1], and the transformed matrix is represented by $X^{{}^{\prime }}$. The conversion formula is shown in Equation (5).

(5) $$x_{p,q}^{{}^{\prime }}=\frac{x_{p,q}-\min\left(x_{q}\right)}{\max\left(x_{q}\right)-\min\left(x_{q}\right)}$$

Although the number of articles published by universities can reflect the scientific research ability of universities to a certain extent, due to the different quality of articles and the different levels of journals, the number of articles published by universities cannot accurately reflect the influence of the scientific research of universities, which needs to be further addressed. To scientifically evaluate article quality, the academic circle is mainly discussed from the perspective of bibliometrics and content analysis. Ren Quan’e et al. [25] used statistical and mathematical tools, and quantitatively evaluated paper quality in terms of citation quantity and journal impact factor. Based on machine learning and natural language processing, Wang Lijun et al. [26] carried out research through text similarity. Although there are many evaluation indicators, there is no unified standard, and some indexes often show defects in practice [27].

Considering the cross-influence of indexes, we choosed “journal compound impact factor” as the influencing factor to evaluate the quality of articles. The journal compound impact factor is the ratio of the citations and the total number of papers published in a journal in the last two years, which is an important indicator reflecting the academic influence of a journal [28]. The composite impact factor data of journals were obtained from the China National Knowledge Infrastructure (CNKI) database. After standardization, the composite impact factor was used as the weight multiplied by the normalized paper number to obtain the RI score of journals in terms of the degree of research innovation of universities and institutions. The calculation formula is shown in Equation (6).

(6) $$RI=IF*X^{{}^{\prime }}$$

where IF is the normalized journal compound factor, and $X^{{}^{\prime }}$ is the normalized number of published papers.

#### 3.2.2. Subject Innovation

Subject innovation (SI) is the result of the further accumulation of the research innovation score of university institutions in journals. At present, there are representative journals in each subject area, and these journals are authoritative for the comprehensive evaluation of the development of university disciplines. By accumulating the scores of the university’s research innovation by the representative journals in the subject field, the subject innovation of the university for each year was obtained, which we used to discuss the development of the university’s discipline from the perspective of time series.

$si_{p}\left(t\right)$ represents the score of the degree of discipline innovation of university *p* in year *t*, $ri_{p,q}\left(t\right)$ represents the value of research innovation of university *p* in journal *q* in year *t*, and *n* represents the total number of journals. The accumulation formula is shown in Equation (7).

(7) $$si_{p}\left(t\right)=\sum_{q=1}^{n}ri_{p,q}\left(t\right)$$

The SI score of university *p* is expressed in Equation (8).

(8) $$SI_{p}=\left[si_{p}\left(t_{1}\right)\;\;\;,\;\;\;si_{p}\left(t_{2}\right)\;\;\;,\;\;\;si_{p}\left(t_{3}\right)\;\;\;,\cdots ,si_{p}\left(t_{a}\right)\right]$$

### 3.3. Characteristic Analysis of Papers Published by Institutions

By quantifying data, clustering, and network building, the analysis process was divided into three aspects: numerical distribution, trend, and association network analyses.

#### 3.3.1. Numerical Distribution Analysis

Numerical distribution analysis involved a clustering analysis based on the numerical time series of university discipline innovation. By applying the clustering analysis method to the SI time series, the university institutions with similar temporal changes in the numerical distribution of SI were divided into the same institution cluster, which not only objectively displayed the dynamic changes in the disciplinary development level of each university, but also allowed the comparison of the disciplinary development levels between universities.

The numerical distribution analysis as shown in Algorithm 2 had two main processing procedures: distance matrix calculation and AP clustering. The algorithm needed the numerical time series of SI as the input, then calculated the Euclidean distance matrix between universities according to the Euclidean distance calculation formula, and used the result as the input value of AP clustering. The final result included several university clusters.

Because the numerical distribution of SI of each university cluster over time was similar, the SI of a university in the cluster center represented the numerical distribution of the university’s corresponding cluster. Therefore, by comparing the numerical distribution level of the cluster centers in the corresponding years, the discipline grades of university institutions could be obtained. To facilitate observation, data visualization techniques were applied to display the charts.
**Algorithm 2** Numerical distribution analysis.**Input:** Numerical time series of SI**Output:** Clustered college clusters $C_{1}$1:Substitute SI data into Formula (1) to calculate the Euclidean distance matrix between universities $D_{1}$2:Substitute distance matrix $D_{1}$ into the AP clustering algorithm3:Obtain clustered college clusters $C_{1}$

#### 3.3.2. Trend Analysis

Trend analysis is a clustering analysis based on the subject innovation trend (ST). The trend data were obtained from the standardization of the time series of institutions’ SI. Because the numerical data of the subject innovation reflected the development level of the discipline through the numerical value, the numerical data could not be used to clearly analyze the trend in the development or the change in the discipline in recent years. Therefore, to analyze the trend with regard to the degree of disciplinary innovation of institutions over time from the perspective of the morphological change in the SI, the time series of the SI were standardized. Then, the normalized time series were converted into the data of subject innovation trends. The normalized conversion is shown in Equation (9).

(9) $$st_{p}\left(t\right)=\frac{si_{p}\left(t\right)-\mu_{p}}{\sigma_{p}}$$

where $\mu_{p}$ represents the mean value of time series $si_{p}$, and $\sigma_{p}$ represents the standard deviation of time series $si_{p}$.

Compared with the numerical distribution analysis, the trend analysis as shown in Algorithm 3 had an additional data normalization process. The input was the numerical time series data of the subject innovation, which we converted into the corresponding trend time series data by standardization. Then, based on the ST data, the Euclidean distance of the university objects was calculated, the result was substituted into the AP clustering algorithm, and the clustered university clusters were output.

Through the time-series clustering method, universities with similar changes in subject innovation over time were found. In the clustering results, the numerical value of the subject innovation of institutions belonging to the same cluster sometimes differed, but the change trend was sometimes similar. According to the value’s fluctuation, the discipline development and changes in university institutions in recent years were analyzed.
**Algorithm 3** Trend analysis.**Input:** Numerical time series of SI**Output:** College clusters $C_{2}$1:Input the numerical data of SI, and use Formula (9) to convert the numerical data into the trend data of subject innovation degree ST2:According to the ST data, substitute Formula (1) to obtain the Euclidean distance matrix between college objects $D_{2}$3:Feed the distance matrix $D_{2}$ into the AP clustering algorithm4:Obtain clustered college clusters $C_{2}$

#### 3.3.3. Association Network Analysis

Based on the clustering results of university research innovation, the association network analysis method was used to construct the association network between universities and journals. Institutions usually grade academic journals according to their own conditions, and take academic journals as the basis for evaluating the scientific research achievements of academics in a school. The grading standards of different universities are often different, and the grading results of journals by the same university usually do not change much from year to year. In reality, researchers usually like to publish in certain journals, such as in certain journals with a high level of influence. Is this phenomenon common? In this study, we hypothesized that there is a certain preference that is characteristic of university publications, and verified this hypothesis by establishing an association network.

University research innovation (RI) is based on the journal’s scoring of the number of scientific research achievements of universities. From the perspective of universities, the choice of a journal for submission is related to the journal’s influence level, showing the university’s preference for the journal. Therefore, the numerical value of the research innovation of institutions can reflect the preference of institutions for journals (preference (PR)): the larger the value, the more inclined the university to publish in the journal.

The initial data in Algorithm 4 named of association network analysis constituted the university–journal research innovation matrix (RI), which was clustered by AP clustering based on the Euclidean distance similarity measurement, and several clustered university clusters were output. By adding the RI scores of each journal to the clustered university clusters, the preference degree (PR) of each university cluster of journals was obtained. By comparing the PR values, the preference characteristics of college clusters for different journals was obtained. If institutions had larger PR values for some journals, then these scholars had preferences when choosing in which journals to publish. In addition, the university–journal association network could be drawn to make the results more intuitive and clear.
**Algorithm 4** Association network analysis.**Input:** Research innovation matrix RI**Output:** College clusters $C_{3}$ and preference degree PR1:Calculate the Euclidean distance matrix $D_{3}$ between institutions2:Substitute the distance matrix $D_{3}$ into AP clustering algorithm3:Obtain the number of college clusters $C_{3}$4:Calculate the total score of each research journal for the research innovation of each university cluster, which is denoted as the preference degree PR of universities to journals5:Compare the PR values

## 4. Empirical Analysis

In this section, the data source is briefly introduced, and the data are processed according to the methods mentioned above. This section’s emphasis is the analysis and discussion of the results.

### 4.1. Dataset

Based on the data of the number of papers published by the double-first-class universities, case studies from the three perspectives were conducted. We took the management discipline as an example. The original data were the published data of 30 important management journals published by the Management Science Department of the National Natural Science Foundation of China, from 42 double-first-class universities from phase *t* tot + 15 (we assumed that the initial phase of time series was *t*). The dataset was mainly obtained from CNKI. CNKI has the largest literature database in China. By setting specific search conditions (year, name of double-first-class university, and name of research journal), the required amount of published data was obtained, captured, and stored. Because the CNKI database did not include any papers from phase *t* to t+15, to ensure the integrity of the data, we obtained the missing data from the Wanfang database [29].

A two-dimensional table was drawn for the annual data from phase *t* to t+15, for a total of 16 two-dimensional tables were obtained, which we indexed by 42 double-first-class universities and institutions. A total of 30 important management journals were classified as categories, and 16 tables were stored. Taking the data of phase t+15 as an example, Table 1 shows the publications of 42 double-first-class universities in 30 management journals in phase t+15. The rows represent universities, and the columns represent management journals. The grid value indicates the number of publications in the corresponding journal (column) of a certain university (row) in phase t+15.

In Table 1, $U_{1}$, $U_{2}$, …, $U_{42}$ the represent 42 universities, respectively. The universities are Peking University (PKU), Renmin University of China (RUC), Tsinghua University (THU), Beijing University of Aeronautics and Astronautics (BUAA), Beijing Institute of Technology (BIT), China Agricultural University (CAU), Beijing Normal University (BNU), Minzu University of China (MUC), Nankai University (NKU), Tianjin University (TJU), Dalian University of Technology (DUT), Jilin University (JLU), Harbin Institute of Technology (HIT), Fudan University (FDU), Tongji University (TCU), Shanghai Jiao Tong University (SJTU), East China Normal University (ECNU), Nanjing University (NJU), Southeast University (SEU), Zhejiang University (ZJU), University of Science and Technology of China (USTC), Xiamen University (XMU), Shandong University (SDU), Ocean University of China (OUC), Wuhan University (WHU), Huazhong University of Science and Technology (HUST), Central south University (CSU), Sun yat-sen University (SYSU), South China University of Technology (SCUT), Sichuan University (SCU), University of Electronic Science and Technology of China (UESTC), Chongqing University (CQU), Xi’an Jiaotong University (XJTU), Northwestern Polytechnical University (NWPU), Lanzhou University (LZU), National University of Defense Technology (NUDT), Northeastern University (NEU), Zhengzhou University (ZZU), Hunan University (HNU), Yunnan University (YNU), Northwest A&F University (NWAFU), and Xinjiang University (XJU). In addition, J1, J2,…, J30, respectively, represent 30 journals. They are the *Journal of Management Sciences in China, System Engineering Theory and Practice, Management World, The Journal of Quantitative & Technical Economics, China Soft Science, Journal of Financial Research, Chinese Journal of Management Science, Journal of Systems Engineering, Accounting Research, Journal of Systems & Management, Business Review, Journal of Industrial Engineering and Engineering Management, Nankai Business Review, Science Research Management, Journal of the China Society for Scientific and Technical Information, Journal of Public Management, Journal of Management Science, Forecasting, Operations Research and Management Science, Studies in Science of Science, China Industrial Economics, Issues in Agricultural Economy, Chinese Journal of Management, Industrial Engineering and Management, Systems Engineering, Science of Science and Management of S.&.T., R&D Management, China Population, Resources and Environment, Journal of Applied Statistics*, and *Management and Chinese Rural Economy*.

According to the method mentioned above, the number of published papers was preprocessed and converted into a university research innovation degree matrix, and a university subject innovation degree numerical and trend time series, which we used for the numerical distribution, trend, and association network analyses of double-first-class universities.

### 4.2. Numerical Distribution Analysis

**Figure 2:**
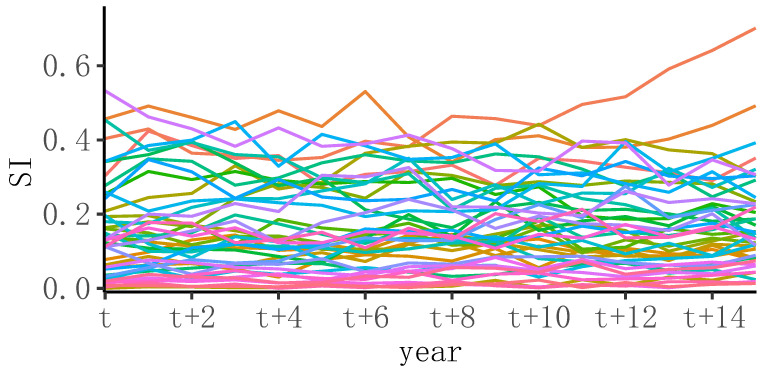
Numerical time series of subject innovation in management disciplines.

Through the numerical distribution analysis method, the numerical distribution of the development of management disciplines in double-first-class universities was analyzed. As shown in Figure 2, the numerical distribution of the degree of research innovation of double-first-class universities in the management discipline was contained within [0, 0.71]. Notably, the distribution was not uniform, and the density characteristic of the time series polyline showed that there were few high-level management disciplines and institutions, while the general and lower-level universities and institutions were numerous. According to the Euclidean distance, the similarity matrix of the time series data was obtained, and AP clustering was performed on the similarity matrix. A total of seven clusters were obtained.

**Figure 3:**
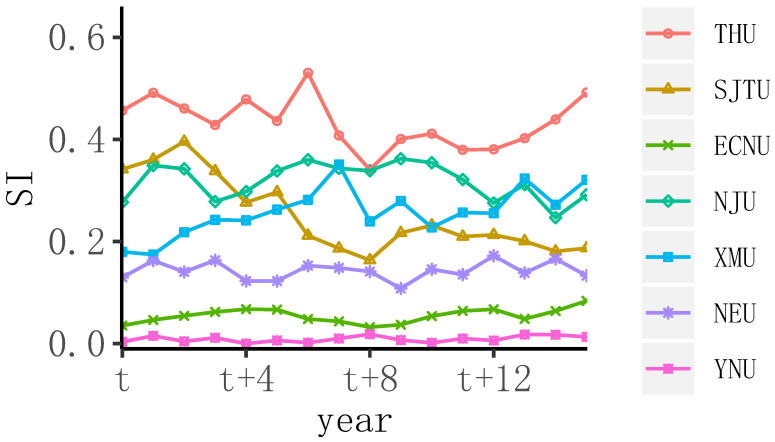
Time series of subject innovation of the cluster center member in the management discipline.

Figure 3 is the time-series cluster center line chart of the clustering results. To analyze the difference in the scientific research level of the management discipline in the double-first-class universities, the cluster center research innovation time-series data were drawn as a line chart, and the scientific research levels of the management disciplines of the seven university clusters were compared. Figure 3 shows that there was a small amount of intersection between the broken lines, which reflects the characteristics of dynamic changes in the development of university disciplines. By grading each year’s disciplinary innovation, 16 grading plans were obtained, showing the development level of management disciplines in these institutions in recent years. The subject innovation of Shanghai Jiaotong University (SJTU) continuously declined in general, which is not conducive to SJTU’s development. Since the t+12 phase, Tsinghua University’s (THU’s) subject innovation has gradually increased, and the scientific research level of such universities has continuously improved, which deserves more attention from the public. The time series with subject innovation as the index can be used to measure the development of scientific research in institutions.

Taking phase t+15 as an example, the development level of management disciplines in double-first-class universities was divided into A++, A+, A, A-, B+, B, and C+, from high to low. There were seven levels, whose corresponding cluster centers were as follows: Tsinghua University, Xiamen University, Nanjing University, Shanghai Jiaotong University, Northeastern University, East China Normal University, and Yunnan University.

Table 2 lists all cluster member universities in order of cluster rank. As shown in Table 2, the cluster at the A++ level centered on Tsinghua University, and the cluster members are Renmin University of China, and Xi’an Jiaotong University; the A+ level clustered at Shanghai Jiaotong University, and the cluster members are Fudan University and Zhejiang University. Most of the top-ranked universities were comprehensive humanities and social sciences universities, such as Renmin University of China. However, the most of the bottom-ranked universities were double-first-class universities with distinctive disciplines, such as Minzu University of China, National University of Defense Technology, Electronics University of Science and Technology, etc. The results showed that the comprehensive institutions with partial humanities and social sciences coverage generally have a stronger research ability in management disciplines than universities with special disciplines. For management students, comprehensive institutions should be prioritized.

**Figure 4:**
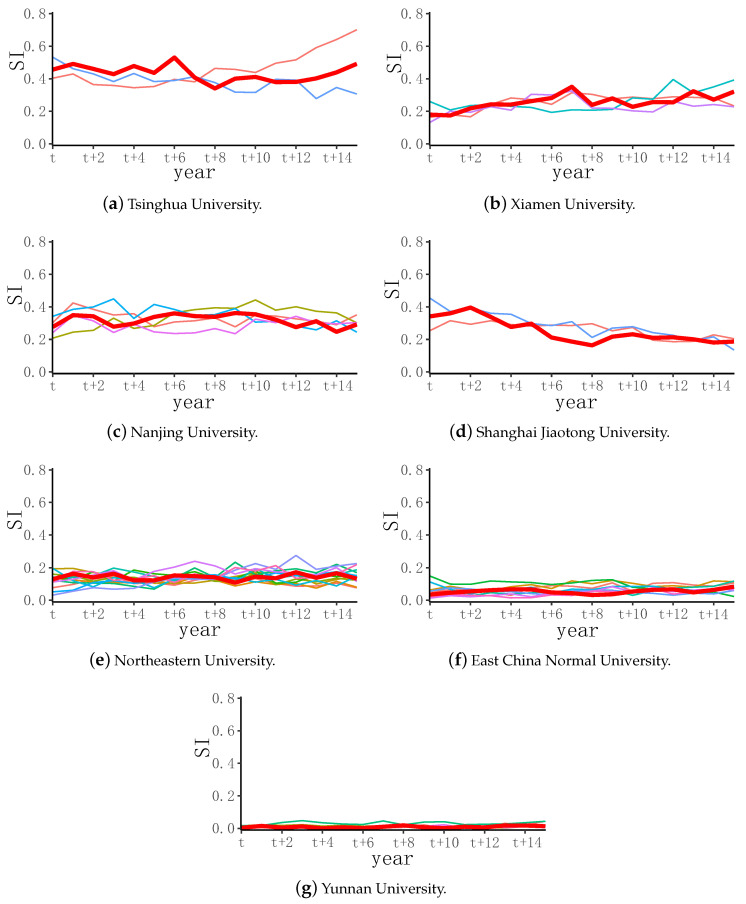
Numerical cluster distribution of SI in management disciplines.

Figure 4 is a broken line chart showing the numerical details of the innovation degree of cluster subjects in each cluster of institutions, in which the cluster centers are represented by bold red lines. The clustering method classified similar development experiences of institutional management disciplines into one category. The broken line shows that the value range of subject innovation between each type of university in the past 6 years was similar, and the development and change in management discipline were also similar. Figure 4a shows the numerical distribution of the innovation degrees of cluster disciplines centered on Tsinghua University. There were a large number of institutions at the A, A-, and B levels. However, most of such institutions’ subject innovation generally failed to rise. Although the management subject was only taken as an example in this paper, this issue might reflect that the scientific research level of most institutions has not improved, which warrants the attention of institutions in the future.

### 4.3. Trend Analysis

**Figure 5:**
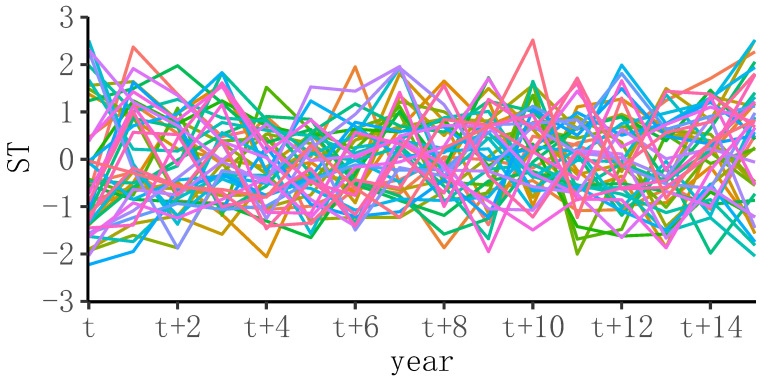
Time series of ST in management disciplines.

Figure 5 shows the overall trend in innovation in management disciplines of 42 double-first-class universities from phase *t* to t+15. The trend steadily fluctuated between [−3, 3], which indicated that, on the whole, there has been little change in the development of the scientific research in the management discipline in double-top universities in the past 16 years. However, from the perspective of individual university objects, the development of the management discipline fluctuated more or less every year.

**Figure 6:**
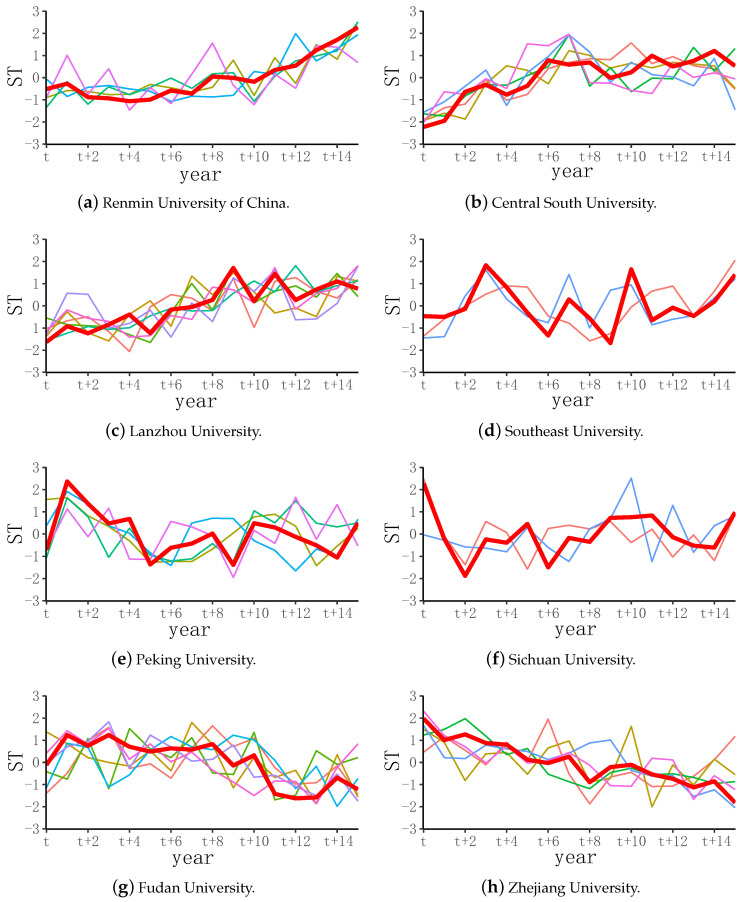
Trend cluster distribution of SI in management disciplines.

AP clustering was carried out on the trend data of SI in institutions, resulting in eight clusters, as shown in Figure 6. The trend changes in each cluster could be classified, which is helpful to strengthening our grasp of the development trend of management disciplines at each institution. Overall, the trends could be roughly divided into three types: ascending (Figure 6a–c), fluctuating (Figure 6d–f), and descending (Figure 6g,h).

Figure 6a is centered on Renmin University of China, and the cluster members include Minzu University of China, Ocean University of China, Wuhan University, and Yunnan University. Since phase t+2 the degree of research innovation of this cluster has shown a steady upward trend. Correspondingly, the discipline level of these institutions has been improving each year. Figure 6b takes Central South University as the center of the cluster, and the cluster members include Nankai University and Dalian. For the University of Science and Technology, Xiamen University, University of Electronic Science and Technology of China, and Chongqing University, the overall degree of research innovation of this cluster of universities has also trended upward, and the development has been relatively flat after t+11. Figure 6c is the cluster with Lanzhou University as the center, including Beijing Institute of Technology, Beijing Normal University, Tongji University, South China University of Technology, Hunan University, and Northwest A&F University. The overall trend in research innovation in this cluster was upward, with obvious fluctuations from phase t+8 to t+12, and a slow rise since phase t+12.

Figure 6d is centered on Southeast University, and the cluster members include East China Normal University and Zhengzhou University. It can be seen that the cluster’s research innovation has significantly fluctuated in recent years. The trend changed from phase *t* to t+13. There has been a significant upward trend in two years, indicating that those universities paid more attention to management disciplines in recent years. In Figure 6e, the cluster is centered on Peking University, including Tianjin University, Sun Yat-sen University, Northwestern Polytechnical University, and Northeastern University. It can be seen from the figure that the trend in research innovation from phase t+4 to t+15 was relatively stable, and the overall trend fluctuated up and down. Figure 6f is centered on Sichuan University, including Shandong and Xinjiang Universities. The cluster was characterized by a sharp decline in the trend from phase *t* to t+2, and the trend gradually stabilized and fluctuated over time.

In Figure 6g, the center of the cluster is Fudan University, and the other members are Beihang University, China Agricultural University, Harbin Institute of Technology, Nanjing University, Huazhong University of Science and Technology, and National University of Defense Technology. Since phase t+12, there has been a slight increase. In Figure 6h, the central university of the cluster is Zhejiang University, and the other members are Tsinghua University, Jilin University, Shanghai Jiaotong University, University of Science and Technology of China, and Xi’an Jiaotong University. The cluster showed significant disciplinary development characteristics.

The development of cluster management disciplines in clustering institutions has varied over time, showing different trends. The innovation degree of the cluster disciplines having Renmin University of China, Central South University, and Lanzhou University as the cluster centers has generally been rising. Combined with the time-series numerical data analysis of the innovation degree of the disciplines, most institutions had a lower ranking in management disciplines, which showed that these institutions have been striving to improve the scientific research level in management disciplines, and have made good progress. There are also some institutions with high cluster rankings with continually rising development trends, such as Renmin University of China, Wuhan University, etc. These high-ranking institutions have continued to develop advantageous disciplines, improve their own discipline level, and work hard toward the goal of building world-class disciplines. Conversely, the innovation degree of clusters centered on Fudan University and Zhejiang University generally showed a downward trend, and the overall trend of clusters centered on Peking University, Southeast University, and Sichuan University showed fluctuations. Therefore, we suggest that the relevant institutions should rationally adjust their discipline development strategy according to the characteristics of the college, pay attention to the frontier academic trends in management disciplines, and comprehensively improve their scientific research capabilities.

### 4.4. Association Network Analysis

**Figure 7:**
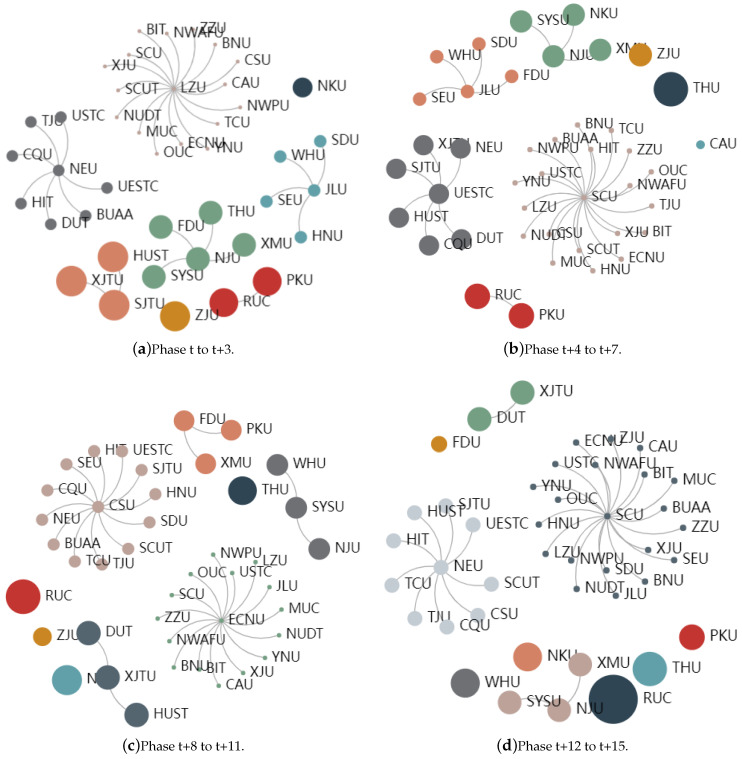
Clustering results of university–journal RI in four periods, from phase *t* to *t* + 15.

The total data were divided into four stages in four-year units, namely, phase *t* to *t* + 3, phase *t* + 4 to *t* + 7, phase *t* + 8 to *t* + 11, and phase *t* + 12 to *t* + 15. By clustering the value of the research innovation degree at each stage, a cluster of institutions with similar preference characteristics to some journals was obtained. The clustering result is shown in Figure 7, where the size of the circle reflects the degree of preference for a journal in the cluster. As shown in Figure 7a, Renmin University of China and Peking University are divided into a cluster, and the circle of the cluster is large; therefore, the two universities had similar characteristics in terms of publishing articles in certain journals, showing a preference for publishing in specific journals. Observing Figure 7, we found that the clustering results of the four time periods were constantly changing, but the changes were not large, which indicated that there was a small change in the preference characteristics of institutions over time. Some institutions, such as Tsinghua University, Zhejiang University, Shanghai Jiaotong University, Huazhong University of Science and Technology, Xi’an Jiaotong University, and Nanjing University, maintained a high degree of preference for certain journals, while other universities, such as Lanzhou University, Yunnan University, and Sichuan University, had a strong preference for these journals. The preference characteristics for management journals were not obvious.

**Figure 8:**
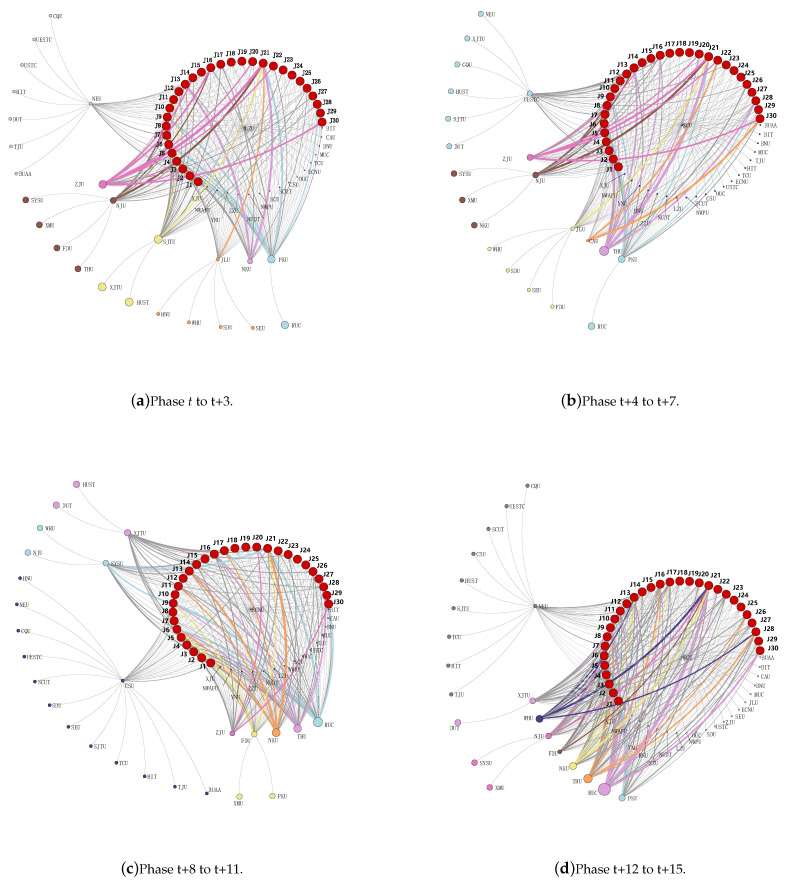
University–journal association network map in four periods from phase *t* to t+15.

The associative network diagram shown in Figure 8 represents the clustering results of each stage, which are connected to the clustered universities and the links between the universities in the cluster centers and journals. The thickness of the connection between the colleges and journals reflects the preference of the cluster of institutions for the journal. To facilitate the observation of the characteristics of the colleges and journals, the connection between colleges and journals with a high degree of correlation is rendered with the color, representing the college cluster. In Figure 8, the line starting from the cluster center and ending with important journals in the management category reflects the cooperative relationship between the college and the journal: the thicker the line, the stronger the preference of the college cluster for the journal.

Taking the period from phase t+12 to t+15 as an example, a more in-depth analysis of the association network between universities and important management journals under the double-first-class construction was carried out. From Figure 8d, ten clusters were obtained from phase t+12 to t+15. The cluster centers are: Peking University, Renmin University of China, Tsinghua University, Nankai University, Fudan University, Nanjing University, Wuhan University, Xi’an Jiaotong University, Northeastern University, and Sichuan University. Peking University, Renmin University of China, Tsinghua University, Nankai University, Fudan University, and Wuhan University are independent clusters, and have obvious associations with individual journals. Taking Renmin University of China cluster as an example, during the four-year period, the researchers at Renmin University of China had a stronger preference for the journals *China Industrial Economics, China Rural Economy, System Engineering Theory and Practice*, and *Accounting Research*. It can be seen that the researchers at Renmin University of China were more inclined to publish in these journals. However, some university clusters did not have an obvious preference for certain journals. For example, the links between the journals and university clusters with Northeastern University and Sichuan University as the core were relatively thin. Combined with the previous analysis, these two university clusters were mostly located in management. The lower level of the disciplinary scientific research indicated that the disciplinary development level of institutions was related to the characteristics of their preference for journals. Generally, the associations between institutions with a lower disciplinary development level and professional journals were more divergent, with no obvious preference. The publications of institutions with a high disciplinary development level had a preference for certain journals and were more concentrated. In addition, from the perspective of journals, *China Industrial Economics* was favored by most of the double-first-class universities with a high level of scientific research, and *Nankai Business Review* and *Accounting Research* were also favored by some double-first-class universities. Some double-first-class universities were more willing to publish articles in their school-run journals. For example, Nankai University preferred its school-run journal *Nankai Business Review*, and preferred other, similar journals to a lesser extent. Similarly, the preference characteristics of university clusters for journals in the other three periods were analyzed, as shown in Figure 8a–c. Over time, even though the clustering of universities had changed, some universities always preferred certain journals.

The analysis showed that there were some characteristics of publishing papers in management journals by double-first-class universities. Some university researchers tended to publish papers in specific professional journals, exhibiting a certain preference. Most of these double-first-class universities were those with a higher development of management disciplines, while those with weaker development had little preference for journals. In addition, for some institutions that ran journals, their on-campus researchers were more inclined to publish articles in the school-run journals, such as *Nankai Business Review*, founded by Nankai University; *Journal of Systems & Management*, founded by Shanghai Jiaotong University, etc. This could be attributed to the preference for these journals by the researchers of the corresponding school, and the researchers of the school were more aware of the characteristics and requirements of the journals. In addition, some professional journals were more popular with university groups, such as *China Industrial Economics* and *Nankai Business Review*.

## 5. Conclusions

In the context of the double-first-class situation, the characteristics of the time-series obtained from the publications of research institutions will help the relevant institutions to understand the construction of the discipline. Through numerical distribution, trend, and associative network analyses, the changes in the institutional disciplines’ development and feature preferences were explored. The first two analysis methods are mainly based on time-series clustering, which we used to study the development of specific disciplines in institutions. Association network analysis was used to study the characteristics of the relationship between colleges and journals, and to identify colleges and journals with frequent cooperation. We selected the management discipline as an example to apply the proposed method. A total of 30 important management journals are recognized by the National Funding Committee as representative journals of management disciplines. In this work, we studied the publishing characteristics of double-first-class universities over the past 16 years from three new analysis perspectives.

We took management journals as the research object, and the changes in scientific research development in institutions over the selected years were displayed. However, the scientific influence of most universities has not steadily improved. We provided ideas for measuring the influence of institutions. Under the double-first-class construction, the scientific research development of institutions is deserving of more attention, and the method proposed in this paper is in line with the current research trend.

We drew some innovative conclusions: (1) We used the measures of research and subject innovation to conduct research, rather than simply publishing data, which led to the conclusions being scientific and effective. (2) From the perspective of time-series clustering, by dividing clusters of institutions with similar discipline development, we dynamically analyzed the development of and changes in institutional disciplines. (3) The proposed analysis method is suitable for analyses of different time periods, institutions, and disciplines.

The lack of an international literature caused some discrepancies in the results of the subject evaluation. The analysis was based only on the publications from universities and colleges in 30 important Chinese journals of economics and management. Publication data from international journals were not included. These limitations meant that we could only discuss the academic level of institutions from the perspective of Chinese publications.

Comprehensive institutions generally had a better management research ability than those focused on special disciplines, which may have been due to the interdisciplinary nature of the management discipline. In future work, to fully evaluate institutions, journals of other disciplines can be taken as the research object to study the scientific research ability of institutions focused on special disciplines for a specific discipline, or journals of various disciplines can be integrated to comprehensively evaluate the scientific research ability of institutions in all aspects.

## Figures and Tables

**Table 1:** Data on the output of institutions in management journals in phase t+15. The asterisk * represents journal number.

30 Management Journals (J *)																														
	**1**	**2**	**3**	**4**	**5**	**6**	**7**	**8**	**9**	**10**	**11**	**12**	**13**	**14**	**15**	**16**	**17**	**18**	**19**	**20**	**21**	**22**	**23**	**24**	**25**	**26**	**27**	**28**	**29**	**30**
**U1**	1	8	10	2	3	15	1	0	7	0	4	2	9	4	1	1	1	0	2	4	4	2	5	0	0	2	1	6	0	2
**U2**	2	6	29	6	14	13	2	1	7	0	17	1	10	7	3	5	4	1	2	3	10	12	11	1	1	6	2	7	8	18
**U3**	2	8	11	3	6	11	6	0	8	1	11	1	4	21	0	4	0	1	6	26	2	4	5	1	0	17	5	14	0	4
**U4**	3	7	1	0	0	0	3	2	0	0	3	3	2	1	0	0	1	0	2	2	0	0	1	2	0	0	0	1	0	0
**U5**	1	7	0	0	2	0	1	1	2	0	4	3	2	4	1	0	1	2	5	6	0	0	2	0	1	2	0	0	0	1
**U6**	0	0	0	1	2	2	1	0	0	0	0	0	0	0	0	0	0	0	1	0	0	8	1	0	0	0	0	2	0	9
**U7**	1	1	7	4	5	1	0	0	1	0	1	0	0	0	1	0	0	0	1	3	1	1	0	0	1	1	1	6	0	1
**U8**	0	0	0	0	2	0	0	0	2	0	0	0	0	0	0	0	0	0	0	0	1	1	1	0	0	0	1	0	1	1
**U9**	1	4	9	2	2	6	3	0	1	2	13	0	7	2	3	1	2	5	4	4	6	1	18	1	2	5	1	1	0	0
**U10**	4	2	1	0	0	0	2	1	2	2	5	1	3	2	2	2	1	4	8	1	0	1	3	6	3	1	3	1	0	0
**U11**	3	8	0	0	4	0	3	5	0	8	14	6	2	16	5	0	1	2	15	15	1	0	5	6	4	12	2	2	0	0
**U12**	1	3	1	1	0	0	0	0	0	0	1	0	1	5	9	0	2	1	1	8	2	1	4	0	0	0	4	2	3	0
**U13**	1	1	3	0	3	0	2	0	1	2	7	1	0	3	0	6	1	1	2	1	0	1	3	0	2	2	3	1	0	0
**U14**	1	1	9	3	1	7	4	0	2	2	0	1	1	2	0	3	0	1	0	2	5	1	1	0	0	1	2	0	1	1
**U15**	2	7	2	2	4	0	4	0	0	3	1	3	1	16	1	1	0	5	7	6	0	1	2	14	2	8	1	2	0	2
**U16**	6	4	7	0	2	0	5	0	0	28	4	4	0	0	0	2	3	1	4	2	0	1	2	28	1	3	2	1	1	1
**U17**	0	0	3	2	0	2	3	0	1	3	1	0	0	3	3	0	0	0	0	0	2	0	2	0	1	0	1	0	3	0
**U18**	7	4	5	1	1	1	3	0	5	6	3	3	2	3	22	1	3	1	3	4	6	2	7	1	2	4	1	4	0	2
**U19**	0	4	1	4	2	3	6	7	5	4	1	4	1	3	0	0	1	0	5	2	2	1	2	6	1	1	0	0	1	0
**U20**	1	0	2	0	1	2	0	0	0	1	0	4	1	10	1	1	0	0	1	13	2	4	1	0	0	1	2	2	0	5
**U21**	0	0	0	1	0	0	3	0	0	0	0	0	0	1	0	0	0	0	4	1	0	0	2	0	0	0	0	1	0	0
**U22**	7	6	6	3	2	8	4	0	7	0	2	3	6	3	0	2	2	0	0	2	6	1	1	2	0	0	0	0	4	0
**U23**	0	1	2	0	0	1	2	0	3	0	4	0	2	2	0	1	1	1	0	2	3	1	5	0	1	3	2	3	1	1
**U24**	0	0	2	0	0	1	2	0	3	1	2	0	3	0	0	0	2	0	0	3	0	2	1	2	0	1	2	6	0	0
**U25**	2	4	0	1	15	8	3	1	5	1	9	4	9	5	17	0	3	2	0	5	9	0	7	0	2	0	1	6	1	2
**U26**	2	5	3	2	1	3	6	0	2	3	7	8	4	6	0	1	4	1	6	4	0	2	13	2	1	1	0	4	0	0
**U27**	1	6	0	1	4	0	6	4	1	2	6	2	0	2	0	0	0	1	10	2	1	3	7	1	5	0	3	4	0	0
**U28**	9	10	11	0	0	7	7	0	4	1	5	4	4	3	2	2	1	1	0	0	5	0	4	0	1	1	5	2	0	0
**U29**	3	7	0	0	3	2	4	1	0	7	7	2	4	12	0	2	0	2	6	10	3	0	8	4	1	6	5	2	0	0
**U30**	0	2	0	1	1	0	1	0	1	0	3	2	0	0	2	1	2	0	0	0	1	1	4	0	0	0	0	3	0	0
**U31**	0	4	0	0	2	0	7	1	2	4	2	1	2	4	1	0	1	1	14	3	0	0	7	6	1	0	1	0	2	0
**U32**	2	7	1	1	3	2	15	1	3	4	11	8	1	6	0	0	0	3	1	0	1	0	5	0	0	1	3	8	2	1
**U33**	1	10	0	4	1	2	8	2	1	7	13	9	3	9	0	0	3	7	18	8	2	1	11	9	3	8	2	3	0	0
**U34**	0	4	0	0	1	0	2	1	0	1	2	7	0	1	0	0	0	1	6	2	0	0	2	2	2	0	1	1	0	0
**U35**	0	1	0	1	1	0	1	0	0	0	2	1	1	3	0	1	0	0	0	0	0	1	1	0	0	1	0	6	1	0
**U36**	0	4	0	0	0	0	0	0	0	0	0	0	0	0	0	1	0	0	1	0	0	0	0	1	0	0	0	1	0	0
**U37**	1	7	2	0	1	0	8	4	1	8	5	0	1	1	0	0	2	1	12	2	0	1	4	5	2	1	1	2	1	0
**U38**	1	2	3	0	1	0	2	0	0	0	0	0	1	1	0	0	0	1	2	1	1	0	0	0	0	0	0	1	0	1
**U39**	3	4	3	2	7	2	7	0	5	6	6	2	3	3	1	0	1	0	2	6	2	2	5	0	1	0	0	3	1	0
**U40**	0	1	0	1	1	0	0	0	0	0	0	0	0	1	0	0	0	0	0	0	0	0	1	0	0	0	0	0	0	0
**U41**	0	0	1	2	0	0	0	1	0	0	1	0	0	0	0	0	0	1	1	0	0	6	0	0	0	0	0	6	0	6
**U42**	0	0	0	0	0	0	0	0	0	0	0	0	0	1	0	0	0	0	0	2	0	0	0	0	1	0	1	1	0	0

**Table 2:** Classification of SI of management discipline in double-first-class construction universities.

Grade	Cluster left	Cluster Member Colleges
A++	THU	RUC, XJTU
A+	SJTU	FDU, ZJU
A	ECNU	BIT, BNU, USTC, OUC, SCU, NWPU, LZU, NWAFU
A-	NJU	PKU, NKU, HUST, SYSU
B+	XMU	DUT, WHU, CQU
B	NEU	BUAA, CAU, TJU, JLU, HIT, TCU, SEU, SDU, CSU, SCUT, UESTC, HNU
C++	YNU	MUC, NUDT, ZZU, XJU

## Data Availability

Not applicable.
